# Development of curcumin‐loaded nanoemulsion stabilized with texturized whey protein concentrate: Characterization, stability and in vitro digestibility

**DOI:** 10.1002/fsn3.3860

**Published:** 2023-11-22

**Authors:** Morteza Jafari, Karim Parastouei, Sepideh Abbaszadeh

**Affiliations:** ^1^ Health Research Center, Life Style Institute Baqiyatallah University of Medical Sciences Tehran Iran

**Keywords:** curcumin, delivery system, extrusion cooking, nanoemulsion, whey protein concentrate

## Abstract

The impacts of pH (2.8, 4.5, and 7.2) and extrusion cooking temperature (60°C, 85°C, and 110°C) on properties of native whey protein concentrate (NWPC) were evaluated, followed by delivering of curcumin through a nanoemulsion system stabilized with extruded WPC (EWPC). Protein solubility, surface hydrophobicity, and emulsion properties such as emulsion activity index (at 1% [w/w] protein concentration), stability index (at 0.5%, 1%, 2%, and 4% [w/w] protein concentration) and creaming index (evaluated at different protein concentrations [0.5%, 1%, 2%, and 4% w/w] and oil levels [20%, 40%, 60%, and 80%]) were improved as a function of the extrusion process. It was found that both covalent and non‐covalent interactions contributed to the stabilization of the extrudates. The rheological investigation of the emulsions stabilized with EWPC (at different oil levels [20%, 40%, 60%, and 80%]) revealed high viscosity and shear thinning behavior as well as much higher *G*′ and *G*″ values. Encapsulation efficiency was increased from 90.8% to 95.7% when NWPC and EWPC were used, respectively. The curcumin‐loaded nanoemulsion containing EWPC presented high stability in confronting with ionic strength (NaCl salt with a concentration of 0.1–1 M), pH (3, 5, and 7), thermal treatments (pasteurization at 63°C for 30 min and sterilization at 95°C for 10 min) and storage time (1 month at 4°C and 25°C). In vitro release behavior revealed that samples stabilized with EWPC showed burst release in simulated intestine conditions. However, it was more stable in stomach conditions.

## Graphical abstract

Covalent and non‐covalent bonds were involved in stabilizing the structure of extruded whey protein concentrate (EWPC), depending on the pH and temperature. Emulsions stabilized with EWPC showed shear thinning behavior, high apparent viscosity, high *G*′ and *G*″ values, as well as superior resistance to environmental stress. The curcumin‐loaded nanoemulsion stabilized with EWPC showed burst release in simulated intestine conditions while remaining more stable in stomach conditions.

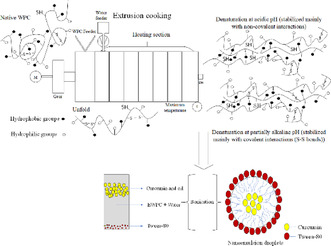

## 1 INTRODUCTION

Curcumin (1,7‐bis(4‐hydroxy‐3‐methoxyphenyl) 1,6‐heptadiene‐3,5‐dione) is a low‐molecular‐weight polyphenolic compound that derives from the rhizome of *Curcuma longa* L. Curcumin has been demonstrated to possess anti‐oxidant, anti‐inflammatory, and antimicrobial properties as well as pharmacological effects against diseases such as Alzheimer's disease, diabetes, and atherosclerosis (Chuacharoen & Sabliov, 2019; Solghi et al., 2020). However, curcumin application is facing challenges due to its poor bioavailability, insufficient solubility in acidic and neutral conditions, poor absorption and rapid metabolism, high hydrolysis rate in neutral and alkaline solutions as well as physiological pH and low resistance to degradation when exposed to light (Ariyarathna & Karunaratne, 2016; Chen et al., 2014; Joung et al., 2016; Mohammadian et al., 2019). Therefore, extensive research has been conducted to overcome these limitations. Different delivery systems were designed to effectively deliver curcumin to targeted sites. To enhance the bioavailability and stability of curcumin, several methods of preparation, such as trapping curcumin inside emulsions and nanoparticles, loading curcumin in gel‐like structures and hydrogel beads, encapsulation of curcumin using coacervate systems and liposomes, complexing curcumin with other ingredients, and incorporating stabilizers, have been used. Among different delivery systems, encapsulation through nanoemulsion systems offers high kinetic stability because of their small droplet size. This system could deliver both hydrophilic and lipophilic phytochemicals. Moreover, the nanoemulsion delivery system provides high bioavailability and enhances the transportation of bioactive compounds through cell membranes (Chuacharoen & Sabliov, 2019; Joung et al., 2016; Li et al., 2014; Sari et al., 2015).

Developing a whey protein (WP) based nanoemulsion is promisingly increasing because WP as a cost‐effective and safe carrier can interact with curcumin via hydrophobic interactions and hydrophilic media simultaneously, improving its dispersity, bioaccessibility, and resistance to environmental stresses (Chen et al., 2014; Li et al., 2014; Mohammadian et al., 2019; Solghi et al., 2020). A number of studies have demonstrated that WP complexes enhance anticancer activity (human colon cancer, SW480, and prostate cancer, LNCap) as well as antioxidant and photostability properties (Jayaprakasha et al., 2016; Mohammadian et al., 2019). Furthermore, McClements (2017) and Solghi et al. (2020) suggested that the increased bioavailability, chemical stability, and photostability of curcumin in nanoemulsions can be attributed to proteins acting as strong antioxidants. The WP performs functions, such as gelling, emulsification, and foaming. WP as an emulsifier adsorbs at the oil–water interface reducing surface tension and limiting the impact of destabilizing phenomena like coalescence, flocculation, and Ostwald ripening (Dickinson & Parkinson, 2004). The properties of emulsions stabilized with WP depend on protein conformation, particle size, degree of denaturation, protein flexibility, water solubility, and the ratio of hydrophilic and hydrophobic residues (Çakır‐Fuller, 2015; McClements, 2004; Surh et al., 2006). A major drawback of whey protein concentration (WPC) for encapsulating lipophilic components such as curcumin is its globular structure. This buries the hydrophobic regions inside, reducing curcumin encapsulation and stability. Furthermore, WP is prone to denaturation/aggregation at different rates depending on processing conditions, such as primary protein concentration, temperature, pH value, and ionic strength (Nicolai et al., 2011). Therefore, applying heat treatments to emulsions stabilized with WP can cause fat droplet aggregation/gelation, resulting in emulsion destabilization (Dickinson & Parkinson, 2004; Li et al., 2014; Mustapha et al., 2012). The functionality of WP could be extended by manipulating its structure. Different structures of WP such as randomly shaped aggregates, nano and microparticles, nanotubes, nanofibrils, flexible strands, and microgels can be formed depending on process conditions such as pH, temperature, moisture content, ionic strength, protein concentration, shear rate, and pressure (Çakır‐Fuller, 2015; Nicolai et al., 2011; Vahedifar et al., 2018).

Extrusion cooking is a type of microparticulate treatment commonly used to achieve controlled unfolding and aggregation as a consequence of applying heat and shear forces simultaneously (Afizah & Rizvi, 2014; Javad et al., 2019; Manoi & Rizvi, 2009; Mozafarpour et al., 2019). The extrusion process weakens the forces involved in stabilizing the tertiary and quaternary structures of the proteins and thus makes them unfold and align with the material flow toward the die (Afizah & Rizvi, 2014; Manoi & Rizvi, 2009; Mustapha et al., 2012). As a result of this process, hidden reactive hydrophobic and thiol groups inside WP's globular structure are exposed, resulting in protein aggregation through non‐covalent interactions and thiol‐disulfide exchange reactions (Afizah & Rizvi, 2014; de la Fuente et al., 2002; Manoi & Rizvi, 2009). pH and temperature have a significant impact on extrudate and aggregate properties. WP is mainly composed of β‐lactoglobulin (β‐lg) and α‐lactalbumin (α‐la). Likewise, the properties of those fractions predominantly govern protein functional properties. Generally, unfolding and conformational changes of α‐la and β‐lg occur at or above 50°C and 80°C, respectively, thereby affecting the emulsifying properties. Actually, exposing hydrophobic patches improves hydrophobic interactions between adjacent proteins and thus creates a rigid film on the surface of oil droplets (Mitidieri & Wagner, 2002). On the other hand, pH influences the type and stability of interactions during aggregation as well as protein solubility. At pH near the isoelectric point (pI), proteins can easily interact with each other and form aggregates due to fewer charges on their surface. While at pH values above and below the pI, proteins have negative or positive charges, respectively, so they repel each other and interact with the surrounding medium (Afizah & Rizvi, 2014; de la Fuente et al., 2002; Javad et al., 2019). Alkaline pH promotes S–S and H–H bonding in proteins and reduces their water solubility while acidic pH increases water solubility, thus affecting protein functionality (Afizah & Rizvi, 2014; de la Fuente et al., 2002; Javad et al., 2019). In general, proteins have a minimum solubility in the pH range of around four to six due to their isoelectric points (pI). The electrostatic repulsion between protein molecules near the isoelectric point is relatively low. This allows them to easily form van der Waals, hydrophobic, or hydrogen bonds, leading to particle aggregation. In contrast, proteins' solubility increases when pH moves away from their isoelectric point as their net charge and electrostatic repulsion become stronger (de la Fuente et al., 2002; Dissanayake et al., 2012).

To the extent of our knowledge, curcumin encapsulation in nanoemulsions stabilized with extruded WPC has not been studied. To this end, the current study evaluated (a) the effect of pH and temperature on the physicochemical, rheological, and emulsification properties of WPC; (b) the use of extruded WPC to deliver curcumin using ultrasound‐assisted nanoemulsion technology; and (c) the evaluation of stability parameters, encapsulation efficiency and release behavior of curcumin under simulated gastrointestinal conditions.

## 2 MATERIALS AND METHODS

### 2.1 Materials

Commercial whey protein concentrate (WPC) was obtained from Kalleh Dairy Ltd. (Amol, Iran). WPC had 81.2 g/100 g protein (dry basis), 5.3 g/100 g fat, 6.6 g/100 g lactose, and less than 3.0 g/100 g ash. Medium‐chain triglycerides (MCT‐60 with predominantly MCT C8 [Caprylic acid; 58.6%] and C10 [Capric acid; 41.4%]) were purchased from Kamani Oil Industries Pvt. Ltd. (Mumbai, India). Curcumin (95% purity), pepsin (activity of more than 3000 units/mg), and pancreatin (200 units/mg) were obtained from Bio Basic Inc. (Canada). Tween‐80 and other chemicals used in this study were purchased from Merck (Darmstadt, Germany) and Sigma‐Aldrich. All chemicals were analytical grade.

### 2.2 Texturization of whey protein concentrate

The WPC powder was preconditioned overnight (10 g/100 g moisture) at room temperature, followed by a texturization process using a co‐rotating twin‐screw extruder (DS56, Jinan Saxin, China) with a length‐to‐diameter ratio of 10:1, a circular opening die with a diameter of 4 mm, screw speed of 300 rpm and feed rate of 35 kg/h. The pH of the samples was adjusted to 2.8, 4.5, and 7.2 using HCl (15%, v/v) and NaOH (5%, w/v) solutions. Accordingly, diluted solutions of HCl and NaOH were sprayed directly onto the powder, followed by the preparation of a 10% (w/w) solution of WP powder to measure the pH. The whey protein powder was continuously stirred during the spraying process. The samples were extruded at die temperatures of 60°C, 85°C, and 110°C under 60% (dry feed basis) moisture content. The samples were dried in an oven at 40°C for 20 h to achieve 5–6 g/100 g moisture. They were ground using a mill machine (Thomas‐Wiley Mill model ED‐5; Arthur H. Thomas Co., PA, USA) and passed through a 60 μm sieve. Based on the following assumptions, the extrusion temperature was selected: below denaturation temperature of β‐lactoglobulin and near to denaturation temperature of α‐lactalbumin (60°C); near to denaturation temperature of β‐lactoglobulin (85°C) and higher than denaturation temperature of β‐lactoglobulin (110°C). The pH conditions were also chosen based on the protein's isoelectric point (pI ~ 4.6).

### 2.3 Particle size distribution and polydispersity index (PDI) of NWPC and EWPC powder

The effective diameters and PDI of native and extruded protein particles were determined in water dispersion (0.1%, w/w) using 90 Plus Particle Size Analyzer (Brookhaven Instruments Corporation, Holtsville, NY, USA), which was calibrated beforehand using a standard solution with particles of known uniform size (Manoi & Rizvi, 2009). The instrument was equipped with MAS OPTION software for particle sizing. In each sample, the intensity‐weighted effective diameter (average diameter) and PDI were determined. Measurements were done in triplicate at room temperature.

### 2.4 Surface hydrophobicity (H_0_)

The surface hydrophobicity of NWPC and EWPC was determined in different buffer solutions (pH 2.8, 4.5, and 7.2) according to the fluorescent probe, 8‐anilino‐1‐naphthalenesulfonic acid (ANS) method (Mustapha, 2013; Mustapha et al., 2012). RF‐540 PC spectro‐fluorometer (Shimadzu Corp., Kyoto, Japan) was used for recording fluorescence intensity at 390 and 470 nm for excitation and emission wavelengths. The index of surface hydrophobicity was determined by the initial slope of the fluorescence intensity (corrected) versus the protein concentration plot using buffer solution of similar pH.

### 2.5 Protein solubility

Protein solubility of NWPC and EWPC was calculated according to the method of Afizah and Rizvi (2014) and Queguiner et al. (1992) with some modifications. Different buffer solutions including (a) deionized water, (b) standard buffer consisting of 0.086 M Tris, 0.09 M glycine, and 4 mM ethylenediaminetetraacetic acid disodium salt (Na2EDTA), pH 8.0, (c) standard buffer containing 8 M urea and 0.5 g/100 g (17.3 mM) sodium dodecyl sulfate (SDS), pH 8.0, and (d) standard buffer containing 8 M urea, 0.5 g/100 g SDS, and 10 mM dithiothreitol (DTT), pH 8.0 were used for extraction of protein. Protein dispersions were centrifuged at 19,000 × g for 15 min at ambient temperature, followed by adjusting to 0.1% w/v. The protein content of supernatant (soluble protein) and dispersion (total protein) was measured using a bicinchoninic acid (BCA) protein assay kit (Thermo Fisher Scientific Inc., Rockford, IL, USA). Protein solubility is defined as the ratio of the protein content of the supernatant to the total protein content of the dispersion.

### 2.6 Emulsifying activity index (EAI) and stability index (ESI)

The protein dispersions were prepared in 0.1 mol/L phosphate buffer adjusted to pH 7. Accordingly, oil in water emulsions based on NWPC and EWPC were prepared by mixing 10 mL MCT oil in 40 mL of protein dispersion, followed by high‐shear mixing using Ultra Turrax T25 basic (IKA Works, Inc., Wilmington, NC, USA) at 22,000 rpm for 2 min. The EAI and ESI of the samples were determined by turbidimetry (Dybowska, 2011; Javad et al., 2019). The EAI was determined at a protein concentration of 1% w/w. In the case of ESI, the impact of different protein concentrations (0.5%, 1%, 2%, and 4% w/w) was investigated.

### 2.7 Creaming index

The creaming stability of NWPC and EWPC emulsions at different protein concentrations (0.5%, 1%, 2%, and 4% w/w) and oil levels (20%, 40%, 60%, and 80%) was evaluated (Javad et al., 2019; Manoi & Rizvi, 2009). Ten milliliter of each emulsion was filled into a glass vial of 6 × 2 cm size and stored at ambient temperature for 7 days. The creaming index was measured by the following formula:

(1) $$\text{Creaming Index}\;\left(\%\right)=\frac{H_{s}}{H_{t}}\times 100,$$

*H*_s_ represent the height of the serum and *H*_t_ shows the total height of emulsions after 7 days.

### 2.8 Rheological characterization of emulsions

According to the previous results, EWPC at 85°C and pH 2.8 (EWPC‐85‐2.8) were selected and the rheological behavior of emulsions based on NWPC and EWPC (4% [w/w] protein concentration) at different oil levels (20%, 40%, 60% and 80%) was measured. The strain‐sweep test was conducted at a frequency of 1 Hz (20°C) and a strain range of 0.01%–100% in order to determine the linear viscoelastic range. Based on the results, 0.2% strain was selected as the linear viscoelastic range (data not shown). The steady shear viscosity parameters and small amplitude oscillatory shear measurements were recorded using a rheometer MCR 301 (Anton Paar GmbH, Germany) equipped with a cup and bob geometry (13.319 mm bob radius, 14.465 mm cup radius, 40.009 mm gap length, 120° cone angle, and 1.146 mm measuring gap).

The apparent viscosity (*η*_a_) was recorded as a function of the shear rate ramping from 1 to 100 s^−1^. The flow curve was fitted to a Power‐law model:

(2) $$\eta_{a}={\mathrm{Ký}}^{n–1},$$

where *η*_a_ is apparent viscosity (Pa.s), *K* is consistency index (Pa.sn), *ý* is shear rate (s^−1^) and *n* is flow behavior index. The viscoelastic properties were determined by a small‐amplitude oscillatory test at frequencies ranging from 0.1 to 100 rad/s. The viscoelastic parameters including elastic modulus (*G*′) and loss modulus (*G*″) were determined.

### 2.9 Preparation and characterization of curcumin nanoemulsion

Curcumin nanoemulsion stabilized with NWPC and EWPC was prepared according to Chuacharoen and Sabliov (2019) and Sari et al. (2015) with some modifications. Curcumin was first dissolved in MCT oil, and then curcumin‐loaded MCT was incorporated into other components of the emulsion. A total of 40 mg of curcumin was present in the emulsion (100 mL), along with 2% (w/w) MCT, 1% (w/w) Tween‐80, and 4% (w/w) WPC. The coarse emulsion was homogenized using a high‐shear mixer, Ultra Turrax T25 basic (IKA Works, Inc., Wilmington, NC, USA), operating at 6500 rpm for 240 s. Then, the fine emulsion was produced using a VCX 130 PB ultrasonic processor (Sonics & Materials, Inc., Newtown CT, USA) with a frequency of 20 kHz, ultrasonic power of 1200 W, power density of 1.36 W/mL, and probe diameter of 20 mm. The sonication amplitude was set at 40% for 10 min. During the sonication process, the temperature was controlled through a water‐cooled jacket.

### 2.10 Particle size distribution, polydispersity index (PDI), and zeta potential of nanoemulsions

The particle size distribution, PDI, and zeta potential of native and extruded WPC stabilized‐emulsions were determined by Zetasizer Nano ZS90 (Malvern Instruments, UK), equipped with dynamic light scattering (DLS) technology. To avoid multiple scattering, the emulsions were diluted with buffer solutions of similar pH before being analyzed. The refractive index of the aqueous phase and oil phase was 1.332 and 1.449, respectively. The particle size was expressed as Dz, intensity‐weighted mean diameter.

### 2.11 Encapsulation efficiency

The encapsulation efficiency was determined according to Chuacharoen and Sabliov (2019) with some modifications. Accordingly, the freshly prepared nanoemulsions were centrifuged at 16,500 × g for 20 min to remove unstable particles and excess curcumin. Curcumin in the precipitate was extracted with 1 mL of ethanol by vortexing for 5 min. The concentration of curcumin (μg/mL) was calculated from the standard curve of curcumin‐ethanol solutions based on its absorbance at 419 nm recorded by a UV–Visible spectrophotometer. The weight ratio of curcumin in the nanoemulsions compared with initial curcumin content was used to calculate the encapsulation efficiency, following the bellow equation:

(3) $$\text{Entrapment efficiency}\;\left(\%\mathrm{EE}\right)=\frac{\text{Entrapped curcumin amount}}{\text{Initial curcumin amount}}\times 100.$$

### 2.12 Determination of retention rate of curcumin

The retention rate of loaded curcumin after being subjected to process and storage conditions was determined by measuring the absorbance of curcumin in extracted solution (extracting with ethanol and vertexing for 5 min) at 419 nm. The retention rate was calculated based on the following equation:

(4) $$\text{Retention Rate}\;\left(\%\mathrm{RR}\right)=\frac{\text{Extracted curcumin}}{\text{Initial curcumin}}\times 100.$$

### 2.13 Stability of nanoemulsion under process conditions

Nanoemulsions were studied under conditions of thermal treatment (pasteurization at 63°C for 30 min and sterilization at 95°C for 10 min), ionic strength (0.1–1 M NaCl), and pH (3–7) and storage for 30 days at 4°C and 25°C. The Dz, PDI, and zeta potential of nanoemulsions (according to Section 2.10), as well as the retention rate of curcumin were determined.

### 2.14 Release kinetics of curcumin

The physiological conditions of the stomach and small intestine were simulated to evaluate the release behavior of encapsulated curcumin (Liang et al., 2012; Sari et al., 2015). The simulated gastric fluid (SGF) was prepared as follows: an aliquot of 3.2 g pepsin and 2 g NaCl was dissolved in distilled water, and then 7 mL HCl (12 M) was added, more water was added to the volume of 1 L and the pH was adjusted to 2 using HCl (5 M). The curcumin‐loaded nanoemulsion (2.5 mL) was mixed with SGF (20 mL) and incubated at 37°C in a shaker (130 rpm) for 2 h (average duration of gastric ingestion transit). The entire sample was divided into different test tubes of equal volume. At the end of digestion, the enzyme was deactivated at 90°C/10 min. Afterward, the mixture of different test tubes after every hour was immediately cooled down in an ice bath (20°C) and centrifuged (5000 × g for 30 min) to adequately separate insoluble‐undigested curcumin. Then, the supernatant was frozen and analyzed for phenolics. After gastric digestion, the samples were immediately mixed with 4.5 mL of simulated intestinal fluid (4 mg/mL pancreatin and 25 mg/mL bile salts) and the mixture (pH 7.5) was then incubated for an additional 3 h at 37°C. The same centrifugation principle was applied to gastric‐digested samples. The total phenolic content of samples was measured after deactivating enzymes, which represents the amount of curcumin released. Total phenolic contents were determined by the Folin–Ciocalteu method based on Singleton et al. (1999) procedure with some modifications. An aliquot of 1 mL of the sample was mixed with 1.5 mL of 7.5% Na_2_CO_3_ and 1 mL of 0.1 M Folin–Ciocalteu reagent. After incubation at room temperature for 2 h in the dark, the absorbance of the reaction mixture was measured at 765 nm using a spectrophotometer (UV‐2600; Shimadzu, Kyoto, Japan) against deionized water blank. The total phenolic content was expressed as gallic acid equivalents (GAE) in mg per g sample.

### 2.15 Statistical analysis

Statistical analyses were performed using the one‐way analysis of variance (ANOVA) by SPSS software (version 23; IBM Software, NY, USA) and significant differences (*p* < .05) between the mean values were determined by Duncan's multiple range procedure. In the case of curcumin release kinetics, the slope of linear regression lines is used to determine whether there are significant differences in the rate of curcumin release. Each experiment was performed at least three times.

## 3 RESULTS AND DISCUSSION

### 3.1 Particle size distribution and PDI of extruded WPC

The extrusion process significantly increased the Dz of WPC (*p* < .05). On the other hand, extrusion processes increased PDI. When the temperature was raised to 85°C, no significant changes were observed among the extrudates (Table 1). In addition, the Dz of these samples did not change significantly when the pH was raised from 2.8 to 7.2, although the Dz decreased slightly when the pH reached 4.5 and increased when the pH was raised to 7.2. The highest Dz and PDI belonged to EWPC‐110‐2.8 and EWPC‐110‐7.2 samples (*p* < .05). Accordingly, the Dz (0.33 μm) and PDI (0.208) of NWPC samples enlarged to 6.94 mm and 0.35 in extruded samples, suggesting a broader distribution of particle sizes. Specifically, the interaction of high temperature (110°C) and pH (far from the isoelectric point of protein) resulted in protein denaturation and unfolding, leading to a significant increase in Dz (*p* < .05). Larger particle sizes reflect increased protein denaturation and aggregation (Afizah & Rizvi, 2014). It seems aggregates created below 85°C are mainly stabilized through weak van der Waals bonding. In contrast, more rigid and dense aggregates were formed when samples were extruded at 110°C. According to previous studies, denaturation of β‐lg at high temperatures is the main cause of WP‐based aggregates and thus larger particle size (de la Fuente et al., 2002; Nicolai et al., 2011). In fact, at higher temperatures (≥90°C) irreversible denaturing and cross‐linking (big aggregates) as well as thiol‐disulfide exchange reactions take place (de la Fuente et al., 2002). In the case of extrudates at 110°C, pH influence was more tangible. This is because increasing pH from 2.8 to 4.5 led to lower Dz values while increasing pH to 7.2 caused larger Dz values (*p* < .05). Similar to our results, Dissanayake et al. (2012) concluded that smaller particle sizes were observed when samples were extruded at lower pH. This was attributed to the cumulative effect of shear and low pH.

**TABLE 1:** Effect of process parameters on Dz, polydispersity and emulsion properties (EAI and ESI) of whey protein extrudates.

Samples	Dz (μm)	PDI	EAI (M^2^/g)‐1%	ESI (min)			
				0.5%	1%	2%	4%
NWPC	0.33 ± 0.02^b^	0.208 ± 0.01^d^	23.48 ± 0.1^e^	30.15 ± 0.2^g^	34.9 ± 0.2^f^	38.29 ± 0.1^f^	47.13 ± 0.3^g^
EWPC‐60‐2.8	3.29 ± 0.01^d^	0.211 ± 0.01^d^	24.9 ± 0.1^b^	31.09 ± 0.3^e^	35.88 ± 0.1^de^	39.18 ± 0.2^de^	55.32 ± 0.4^d^
EWPC‐60‐4.5	3.08 ± 0.02^d^	0.206 ± 0.02^d^	24.14 ± 0.2^d^	30.83 ± 0.2^f^	35.23 ± 0.2^ef^	38.46 ± 0.1^f^	54.29 ± 0.3^e^
EWPC‐60‐7.2	3.38 ± 0.01^cd^	0.231 ± 0.01^c^	24.05 ± 0.1^d^	30.5 ± 0.1^g^	35.07 ± 0.2^f^	38.36 ± 0.1^f^	53.59 ± 0.3^f^
EWPC‐85‐2.8	3.31 ± 0.01^d^	0.227 ± 0.03^c^	25.47 ± 0.1^a^	33.37 ± 0.3^b^	36.73 ± 0.1^c^	39.82 ± 0.1^d^	57.71 ± 0.4^b^
EWPC‐85‐4.5	3.27 ± 0.02^d^	0.214 ± 0.02^cd^	24.39 ± 0.2^cd^	32.65 ± 0.1^cd^	36.08 ± 0.2^d^	38.94 ± 0.3^ef^	56.37 ± 0.3^c^
EWPC‐85‐7.2	3.43 ± 0.02^c^	0.245 ± 0.03^c^	24.17 ± 0.2^d^	32.1 ± 0.1^d^	35.71 ± 0.1^de^	38.58 ± 0.2^f^	55.83 ± 0.5^cd^
EWPC‐110‐2.8	6.82 ± 0.05^a^	0.342 ± 0.03^a^	24.66 ± 0.1^bc^	34.08 ± 0.3^a^	38.1 ± 0.2^a^	56.58 ± 0.2^a^	158.35 ± 0.9^a^
EWPC‐110‐4.5	5.83 ± 0.02^b^	0.302 ± 0.02^b^	23.82 ± 0.1^e^	33.79 ± 0.2^ab^	37.3 ± 0.3^b^	53.24 ± 0.3^b^	157.9 ± 0.8^a^
EWPC‐110‐7.2	6.94 ± 0.06^a^	0.350 ± 0.02^a^	22.97 ± 0.2^f^	32.87 ± 0.1^c^	36.68 ± 0.1^c^	52.6 ± 0.2^c^	157.27 ± 0.9^a^

*Note*: Temperature: 60°C, 85°C, and 110°C; pH 2.8, 4.5, and 7.2; percentage of whey protein concentration: 0.5%, 1%, 2%, and 4%. Means with the same letter in each column are not significantly different (*p* < .05).

Abbreviations: EAI, emulsion activity index; ESI, emulsion activity index; EWPC, extruded whey protein concentrate; NWPC, native whey protein concentrate.

### 3.2 Surface hydrophobicity

**FIGURE 1:**
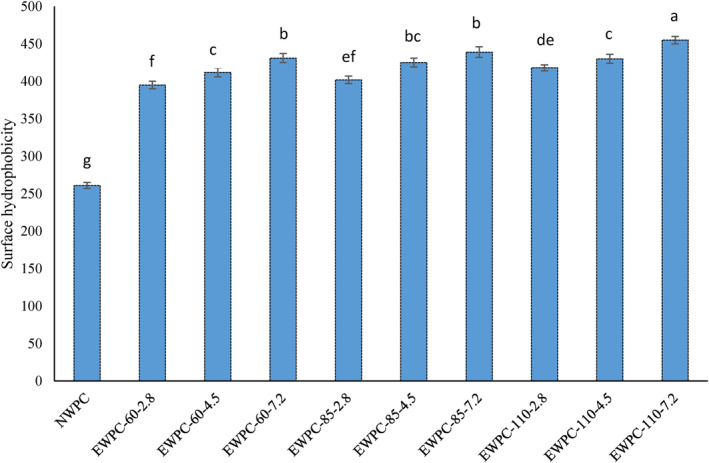
Effect of process parameters on surface hydrophobicity of whey protein extrudates. EWPC, extruded whey protein concentrate; NWPC, native whey protein concentrate; temperature: 60°C, 85°C and 110°C; pH 2.8, 4.5 and 7.2. Different letters in each column denote significant differences between samples (p < .05).

The surface hydrophobicity of a protein refers to the number of non‐polar groups present on its surface when it is in contact with a polar solution (Manoi & Rizvi, 2009; Mustapha et al., 2012). The results showed that the EWPC surface hydrophobicity increased considerably compared to the NWPC (*p* < .05) (Figure 1). Among the extrudates, the lowest surface hydrophobicity belonged to EWPC‐60‐2.8 and EWPC‐85‐2.8 samples, while the highest value was related to EWPC‐110‐7.2 sample (*p* < .05). Increasing temperature up to 110°C caused a continuous increase in surface hydrophobicity. High surface hydrophobicity indicates enhanced ANS accessibility and improved binding to hydrophobic sites on protein molecules. Lower surface hydrophobicity might be attributed to increased intra‐molecular interaction and consequently burial of some hydrophobic groups (Mustapha et al., 2012; Silva et al., 2010). In accordance with our results, Mozafarpour et al. (2019) reported that the surface hydrophobicity of extruded soy protein decreased, which can be attributed to protein–protein interactions. Furthermore, those authors concluded that the partial dissociation of aggregates and protein unfolding led to higher surface hydrophobicity, which may have enhanced the interactions between hydrophobic residues and ANS. However, the burying of some hydrophobic groups inside protein aggregates is considered the main reason for decreasing surface hydrophobicity (Mozafarpour et al., 2019). Denaturation and texturization of WPC after extrusion cooking enhance surface hydrophobicity by exposing the hydrophobic group buried inside the protein molecules (Manoi & Rizvi, 2009). Accordingly, it is stated that above the denaturation temperature of β‐Lg, the surface hydrophobicity of the proteins increases regardless of acidic or neutral pH (Manoi & Rizvi, 2009; Mustapha et al., 2012). Additionally, Silva et al. (2010) suggested that molecular reorganization of proteins and conformation changes of β‐Lg as well as exposure of hydrophobic amino acid groups as a function of shear and temperature is a reason for increasing surface hydrophobicity.

### 3.3 Protein solubility

Solubility is a crucial parameter closely related to functional properties and reflects protein denaturation degree. Solubility indicates whether proteins are native or denatured (Manoi & Rizvi, 2009). The results are given in Table 2. NWPC had the highest solubility (84.32%) (*p* < .05). NWPC's relatively low solubility suggests that some protein is partially denatured during preparation and spray drying. It was found that the extrusion process significantly reduced its solubility (*p* < .05). Increasing the extrusion temperature drastically reduced the solubility, indicating denaturation and aggregation of proteins. The lowest solubility was belonged to EWPC‐110‐7.2 and EWPC‐110‐4.5 samples (*p* < .05). High surface hydrophobicity of extruded samples could contribute to decreasing WPC solubility, as hydrophobic residues improve hydrophobic interactions between proteins over protein‐water interactions. In addition, high surface hydrophobicity enhances aggregate formation because it helps them shield their non‐polar residues and thus results in lower solubility (Afizah & Rizvi, 2014; Mitidieri & Wagner, 2002). The current study was in accordance with Afizah and Rizvi (2014) who reported that increasing extrusion temperature to 90°C led to lower solubility in WPC. Among the extrudates, the highest solubility was observed in samples treated at lower pH (EWPC‐60‐2.8 and EWPC‐60‐4.5) (*p* < .05). Further increases in pH led to lower solubility, which was supported by the hydrophobicity trend.

**TABLE 2:** Effect of process parameters on solubility of whey protein extrudates.

Samples	Solubility (%)			
	Water	Buffer 1	Buffer 2	Buffer 3
NWPC	84.32 ± 0.5^Ca^	86.4 ± 0.3^BCa^	93.36 ± 0.3^ABab^	96.86 ± 0.3^Aa^
EWPC‐60‐2.8	78.5 ± 0.4^Cb^	81.43 ± 0.7^Bab^	95.82 ± 0.5^Aa^	96.76 ± 0.4^Aa^
EWPC‐60‐4.5	75.31 ± 0.3^Bbc^	77.63 ± 0.7^Bbc^	95.43 ± 0.5^Aa^	96.64 ± 0.4^Aa^
EWPC‐60‐7.2	72.4 ± 0.7^Bc^	73.8 ± 0.8^Bcd^	95.53 ± 0.7^Aa^	96.68 ± 0.6^Aa^
EWPC‐85‐2.8	29.24 ± 0.7^Dd^	63.38 ± 0.2^Cde^	95.08 ± 0.6^Ba^	96.92 ± 0.7^Aa^
EWPC‐85‐4.5	24.25 ± 0.6^Dde^	48.23 ± 0.4^Cf^	95.76 ± 0.3^Ba^	96.88 ± 0.6^Aa^
EWPC‐85‐7.2	21.8 ± 0.8^Def^	56.86 ± 0.1^Cef^	95.13 ± 0.8^Ba^	96.88 ± 0.5^Aa^
EWPC‐110‐2.8	19.7 ± 0.4^Cfg^	38.47 ± 0.5^Bgh^	94.18 ± 0.3^Aa^	96.18 ± 0.4^Aa^
EWPC‐110‐4.5	15.79 ± 0.4^Dgh^	33.76 ± 0.8^Chi^	89.17 ± 0.6^Bb^	95.21 ± 0.3^Aab^
EWPC‐110‐7.2	13.65 ± 0.3^Dh^	30.14 ± 0.6^Ci^	48.35 ± 0.4^Bc^	93.93 ± 0.4^Ab^

*Note*: Temperature: 60°C, 85°C and 110°C; pH 2.8, 4.5 and 7.2. Solubility of NWPC and EWPC in water and selected buffers. Buffer 1: 0.086 mol/L Tris, 0.09 mol/L glycine, and 4 mmol/L Na2EDTA, pH 8.0; Buffer 2: 8 mol/L urea and 0.5 g/100 g SDS; Buffer 3: 8 mol/L urea, 0.5 g/100 g SDS, and 10 mmol/L dithiothreitol (DTT). For each sample, different capital letters within the same row indicate significant differences in solubility in water and selected buffers (buffer 1, 2, and 3). Different small letters within the same column indicate significant differences between native and extruded samples in terms of solubility in water and selected buffers (buffer 1, 2, and 3).

Abbreviations: EWPC, extruded whey protein concentrate; NWPC, native whey protein concentrate.

Protein solubility depends on proteins' affinity toward protein–protein or protein–solvent interactions through covalent (disulfide linkages) and/or non‐covalent interactions (hydrogen and hydrophobic bonds) (de la Fuente et al., 2002; Javad et al., 2019; Vahedifar et al., 2018). Therefore, the samples were dissolved in different buffers including EDTA (ionic and hydrogen bonds), urea and SDS (hydrogen bonds and hydrophobic interactions), and DTT (disulfide bonds), to find out the nature and the role of different types of interactions in the stability of the protein structure (Afizah & Rizvi, 2014; Queguiner et al., 1992).

Generally, it was found that the least amount of protein was extracted in a standard buffer containing EDTA (Table 2). The EWPC‐110‐7.2 sample contained the least amount of soluble protein (*p* < .05), likely due to protein aggregation. In contrast, the highest solubility was observed in EWPC‐60‐2.8 and EWPC‐60‐4.5 samples (*p* < .05). Protein aggregation and protein solubility loss are highly dependent on pH. The higher solubility at pH 2.8 might be attributed to superior thermostability and less aggregation of β‐lg at acidic conditions (Afizah & Rizvi, 2014; Queguiner et al., 1992). The lower solubility at pH 4.5 is due to approaching the isoelectric point and reducing the electrostatic repulsive forces between protein molecules.

The urea and SDS results indicated that regardless of pH changes, the samples extruded at 60°C and 85°C showed approximately 95% solubility, indicating that non‐covalent interactions (hydrophobic interactions and hydrogen bonds) played a primary role in maintaining the structure of these samples. Likewise, the solubility of native whey protein was also increased, implying that non‐covalent interactions contributed to NWPC structure stability. The solubility of samples extruded at 110°C (EWPC‐110‐2.8, EWPC‐110‐7.5, EWPC‐110‐7.2) were 94.18%, 89.17% and 48.35%, respectively, suggesting that non‐covalent interactions played a lesser role in stabilizing the structure of samples produced at pH 7.2. The presence of DTT enabled extrudates to be nearly completely dissolved, especially those prepared under a temperature of 110°C and pH 7.2. A high degree of S‐S bonds could be related to the increased negative charge of proteins. In the current study, we found that protein–protein interactions during extrusion cooking were significantly influenced by pH conditions and temperature.

### 3.4 Emulsion activity index (EAI), emulsion stability index (ESI), and creaming index

EAI refers to proteins' ability to absorb at the interface of oil–water droplets. One of the most prominent features of proteins is their ability to realign themselves between the oil and aqueous phases through hydrophobic and hydrophilic patches to reduce dynamic interfacial tension (Dybowska, 2011; Mitidieri & Wagner, 2002). Therefore, the adsorption velocities of proteins at the interface, the flexibility of their structure, and their ability to conform to conformational changes have a remarkable impact on their emulsification performance (Dybowska, 2011; Javad et al., 2019).

EAI results immediately following emulsion preparation revealed, in general, that after extrusion treatment, EAI increased (Table 1). However, this trend was not very sharp. The highest EAI was belonged to EWPC‐85‐2.8 sample (*p* < .05). However, the lowest EAI was observed for EWPC‐110‐7.2 (*p* < .05). The EAI was raised up to 85°C. However, further increasing the temperature resulted in a lower EAI (*p* < .05). In the case of NWPC, it has been reported that unfolding and uncoiling the secondary structure of globular proteins takes more time (McClements, 2004). Due to this, their ability to re‐arrange and form a stable viscoelastic layer around fat globules is reduced, resulting in reduced EAI. In contrast, improving the surface hydrophobicity and molecular flexibility of extruded samples as well as enhancing their ability to readily realign themselves results in effective adsorption of protein at the oil–water interface and thus higher EAI (Manoi & Rizvi, 2009; Mitidieri & Wagner, 2002). In fact, hydrophobic interactions between adjacent proteins at the interface increase with extrusion cooking (Manoi & Rizvi, 2009). By considering the fact that the surface hydrophobicity of samples extruded at pH 2.8 was less than others (Table 1), it seems that the EAI should be low in these samples, while this trend was not observed. In accordance with this, some authors pointed out that mechanical forces applied by ultrasound and homogenizers could disrupt intermolecular interactions that stabilize aggregates. This could increase the exposure of hydrophobic groups, resulting in increased emulsifying activity (Mozafarpour et al., 2019). Our results were in accordance with Javad et al. (2019) who reported that EAI values of EWPC at pH 3 and 5.4 were higher than NWPC. Moreover, higher EAI was found for samples extruded at pH 3 than pH 5.4.

In order to investigate the effect of protein concentration on emulsion stability upon storage, ESI was evaluated. The results are shown in Table 1. The ESI refers to an emulsion's resistance to coalescence over time. The results showed that extrusion pretreatment remarkably improved emulsion stability. NWPCs have lowest ESI (*p* < .05) due to their rigid packed globular structure (Manoi & Rizvi, 2009; McClements, 2004). It was found that emulsions stabilized with extrudates prepared at higher temperatures and more acidic conditions (EWPC‐110‐2.8) were more stable (*p* < .05). EI and ESI did not show a direct relationship as raising the temperature to 110°C resulted in a higher ESI, while the EAI decreased as the temperature rose. According to Dybowska (2011), there is no direct relationship between ESI and EAI as the viscosity of continuous phases dominates ESI. Additionally, the strength of protein–oil interactions and the nature of protein–protein interactions affect ESI. In fact, the EAI is mainly determined by the balance between surface hydrophobicity and solubility (Afizah & Rizvi, 2014; Dybowska, 2011). The emulsion's stability was increased when the protein concentration was increased (*p* < .05). It could be related to the higher concentration of protein at the interface and the formation of a strong viscoelastic film around the fat globules via non‐covalent intermolecular interactions and covalent disulfide cross‐linking, which enhances the viscosity of the continuous water phase and thickens the protein adsorbed layer surrounding fat globules (Li et al., 2014; Manoi & Rizvi, 2009). According to McClements (2004) and Surh et al. (2006), electrostatic and steric repulsion are the two main stabilizing forces for whey protein‐stabilized emulsions. In addition, greater ESI could be attributed to the higher surface hydrophobicity and molecular flexibility of EWPC samples compared to NWPC samples, since higher surface hydrophobicity contributes to the formation of rigid films through hydrophobic interactions between adjacent protein molecules at the interface and increased molecular flexibility allows effective adsorption of protein (Manoi & Rizvi, 2009). The highest ESI was found for extrudates at 110°C and 4% protein concentration (EWPC‐110‐2.8, EWPC‐110‐4.5, and EWPC‐110‐7.2) (*p* < .05).

**FIGURE 2:**
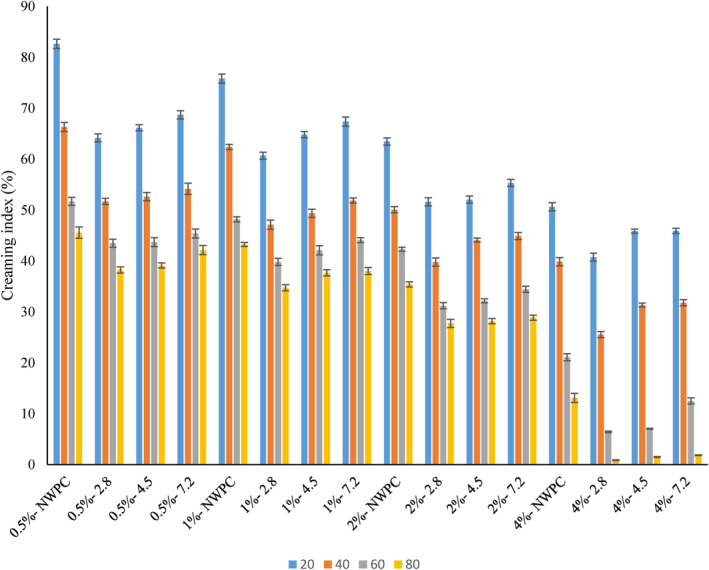
Effect of extruded whey protein concentration on creaming index at different oil and protein level. Samples were extruded at 85°C at different pH 2.8, 4.5, and 7.2. NWPC, native whey protein concentration. The rest of the codes are related to extruded samples. The percentage of protein: 0.5%, 1%, 2% and 4%; the oil level: 20%, 40%, 60% and 80%.

In order to test the impact of oil concentration at different protein levels on the creaming index, samples extruded at 85°C were selected by considering parameters such as higher EAI, surface hydrophobicity, fewer disulfide bonds, and smaller Dz (Figure 2). The creaming index represents the rate of coalescence in which forces such as colloidal, gravitational, hydrodynamic, and mechanical are involved. It indicates the resistance of the viscoelastic layer of proteins to rupture after a period of storage (McClements, 2004). The creaming index was reduced by extrusion cooking. The results showed that the pH of the extrudates had an impressive effect on the creaming index. The samples prepared at pH 2.8 had the lowest creaming index level. Increasing the protein and oil concentration decreased the creaming index. The lowest creaming index for NWPC (13.12%) and EWPC (0.87%) was found at a 4% protein concentration with an 80% oil level. The continuous phase viscosity increases when the oil concentration rises. This restricts oil droplets' movement, resulting in a lower creaming index. In fact, neighboring droplets at higher oil levels reduce droplet velocity and movement. In addition, high protein levels reduce oil droplet coalescence since higher amounts of proteins adsorb at the interface, which would be sufficient to cover oil droplets (Javad et al., 2019; Ma et al., 2017; Manoi & Rizvi, 2009; McClements, 2004). In accordance with our results, Javad et al. (2019) reported that the creaming index of EWPC was decreased. Low creaming index of extruded proteins could be associated with (a) elevated steric and electrostatic hindrance as a result of increasing hydrophobicity; (b) creating a thicker layer of adsorbed proteins on the surface of oil droplets through hydrophobic interactions; and (c) smaller particle size which promotes the viscosity of the emulsion (Afizah & Rizvi, 2014; Ma et al., 2017; Manoi & Rizvi, 2009; McClements, 2004). In contrast to NWPC, aggregated proteins during extrusion cooking form a thicker layer when adsorbing on the droplet surface. In addition, partial protein unfolding and consequently increasing hydrophobicity promote inter‐droplet protein–protein interactions over intra‐droplet interactions, leading to higher stability and a lower creaming index.

### 3.5 Rheological characterization of emulsion

#### 3.5.1 Steady shear viscosities

**FIGURE 3:**
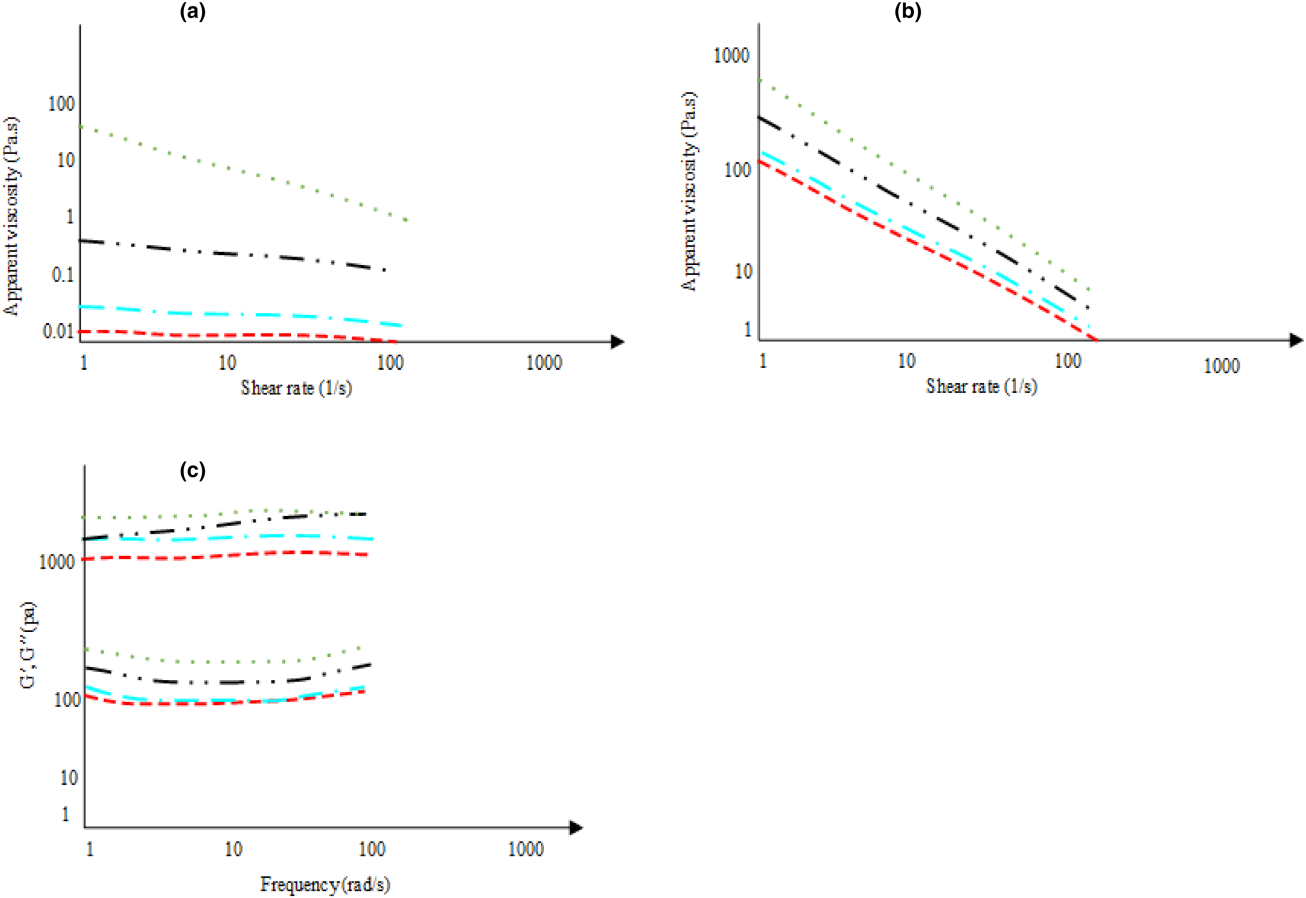
Flow behavior profile of native (a) and extruded (b) whey protein stabilized emulsion at different oil level. Variations in *G*′ (storage modulus, upper lines) and *G*″ (loss modulus, bottom lines) with frequency at 25°C for emulsions stabilized with extruded whey protein at different oil level (20%: red line; 40%: blue line; 60%: black line and 80%: green line) were presented at figure (c).

In order to evaluate high‐protein‐based systems, 4% protein was selected for further analysis. The rheological parameters are shown in Table 3. Generally, all the samples exhibited shear thinning behavior in the shear rate ranges of 1–100 s^−1^, in which EWPC represented a more pronounced shear thinning behavior (*n* = 0.112–0.223) than NWPC (*n* = 0.912–0.958). The emulsions stabilized with NWPC presented almost Newtonian behavior when the oil level was 20%–60% (Figure 3). However, more shear‐thinning behavior was obtained at 80% oil concentration, indicating fat droplet flocculation. In fact, at higher oil levels, a network of flocculated adjacent oil droplets is created which deforms and disrupts as a consequence of the shear rate, leading to lower viscosity (de la Fuente et al., 2002; McClements, 2004). The viscosity versus shear rate plot of an emulsion prepared using EWPC illustrated that a noticeable shear‐thinning behavior was observed at all oil levels. Moreover, the results showed that EWPC's viscosity was considerably higher than that of NWPC. This indicates that EWPC adsorbed on the surface of oil droplets formed a more elastic layer, which enhanced the resistance to rupture during shear. However, a weak elastic layer is formed in NWPC, leading to easy disruption of aggregated oil droplets and thus lower viscosity (Afizah & Rizvi, 2014; de la Fuente et al., 2002; Manoi & Rizvi, 2009). The higher viscosity of emulsion stabilized with EWPC is ascribed to denaturation and unfolding of the globular structure of the protein which led to stronger protein–protein and hydrodynamic interactions and thus the apparent viscosity improved due to higher viscosity of continuous phase (de la Fuente et al., 2002; Manoi & Rizvi, 2009; McClements, 2004). Manoi & Rizvi (2009) claimed that denaturation of protein promotes viscosity because it leads to protein molecular entanglements and protein–protein interactions. Furthermore, the unfolding of protein increases water‐holding capacity via the formation of physical cross‐links and hence increases apparent viscosity (de la Fuente et al., 2002). McClements (2004) and Dissanayake et al. (2012) reported that protein denaturation and consequently conformational rearrangements changed the surface charge which in turn could promote viscosity via enhancing protein–protein interactions. On the other hand, it has been deduced that emulsions with smaller particle sizes generally have higher viscosity, because of the overlapping of double layers present at the interface of smaller particles (de la Fuente et al., 2002; Dissanayake et al., 2012; McClements, 2004). Therefore, nanoemulsions stabilized with EWPC had a higher viscosity than those stabilized with NWPC. These results are supported by increasing the emulsion consistency index. It was proposed that raising the oil level improved the system's consistency, implying that at higher oil concentrations the movement of the oil droplets was restricted and hence resulted in a higher consistency index.

**TABLE 3:** Effect of oil level on the rheological properties of emulsion stabilized with native and extruded whey protein concentration.

Samples/oil level	Power law parameters				Mechanical spectra	
	*η*_a_ (Pa.s)	*n*	*K* (Pa.s^*n*^)	*R* ^2^	*G*′ (Pa)	*G*″ (Pa)
NWPC						
NWPC‐20%	0.01 ± 0.002^g^	0.958 ± 0.08^a^	0.01 ± 0.001^e^	.993	NA	NA
NWPC‐40%	0.05 ± ±0.004^g^	0.935 ± 0.06^a^	0.02 ± 0.002^e^	.995	NA	NA
NWPC‐60%	0.23 ± 0.009^f^	0.922 ± 0.1^a^	0.03 ± 0.001^e^	.997	NA	NA
NWPC‐80%	6.27 ± 0.08^e^	0.912 ± 0.09^a^	0.03 ± 0.004^e^	.994	26.18 ± 0.2^e^	4.86 ± 0.3^e^
EWPC						
EWPC‐20%	9.18 ± 0.09^d^	0.223 ± 0.01^b^	20.68 ± 0.1^d^	.996	433.56 ± 5^d^	42.68 ± 3^d^
EWPC‐40%	12.78 ± 0.11^c^	0.187 ± 0.02^bc^	22.23 ± 0.1^c^	.996	497.29 ± 3^c^	55.23 ± 3^c^
EWPC‐60%	36.67 ± 0.07^b^	0.141 ± 0.01^c^	27.55 ± 0.2^b^	.998	908.60 ± 7^b^	79.55 ± 6^b^
EWPC‐80%	63.45 ± 0.14^a^	0.112 ± 0.03^c^	32.38 ± 0.2^a^	.997	1387.16 ± 15^a^	87.38 ± 5^a^

*Note*: EWPC, extruded whey protein concentration at pH 2.8 and process temperature of 85°C. NA: test was not performed because emulsions were too liquid and unstable. Different letters in the same column denote significant differences between samples (*p* < .05). The mechanical spectra were compared at 1 rad/s.

Abbreviations: *G*′, elastic modulus; *G*″, viscous modulus; *K*, consistency index; *n*, flow behavior index; NWPC, native whey protein concentration; *R* ^2^, determination coefficients; *η*_a_, apparent viscosity.

#### 3.5.2 Viscoelastic properties

The oscillation test was conducted to better understand the relationship between the emulsion's internal structure and its macroscopic properties (Table 3 and Figure 3). At small strains, the microstructure remains intact and thus the viscoelastic properties of the original structure could be investigated. As a result of their poor consistency in the linear viscoelastic range, the viscoelastic properties of emulsions stabilized with NWPC with oil concentrations of 20%–60% were not estimated. At the oil fraction of 0.8, the *G*″ was higher than *G*′ (data not shown), indicating a viscous‐like behavior. These samples showed a high‐frequency dependence in dynamic moduli (data not shown), originating from non‐flocculated or weakly flocculated emulsions.

In the case of emulsions stabilized with EWPC, higher *G*′ and *G*″ were obtained than samples containing NWPC. Frequency‐independent behavior was observed at all frequencies studied. The *G*′ line was always over the *G*″ values, indicating a solid gel‐like structure (Figure 3). Dickinson and Parkinson (2004) concluded that high elastic modulus and gel‐like structure mainly originated from the creation of a network of entangled adsorbed and non‐adsorbed protein molecules.

The *G*′ and *G*″ of both NWPC and EWPC‐stabilized emulsions increased when the oil concentration increased, indicating that dynamic mechanical moduli were dependent on oil concentration. The high *G*′ values observed at high oil levels indicate that emulsions became more solid and gel‐like due to the fact that, at higher oil levels, a compact matrix of oil droplets forms (Mustapha et al., 2012). Manoi & Rizvi (2009) and Mustapha et al. (2012) regarding the influence of EWPC on the rheological properties of concentrated emulsions, have reported similar results.

### 3.6 Changes in characterization of nanoemulsion under different conditions

#### 3.6.1 Effect of ionic strength

After preparing the curcumin‐loaded nanoemulsion, the effect of environmental stresses was investigated. Salts can change the functional performance of proteins by affecting charge balance, surface charge, and electrostatic repulsion. In this regard, the effect of ionic strength (NaCl salt with a concentration of 0.1–1 M) on the particle size and zeta potential of nanoemulsion containing curcumin was evaluated (Table 4). The particle size indicates the tendency of the emulsion toward flocculation or coalescence. Nanoemulsions stabilized with EWPC had smaller particle sizes than those stabilized with NWPC at all ionic strengths. However, no significant differences were observed in terms of polydispersity. It could be ascribed to the higher net charge of the extruded protein, which prevents coalescence of droplets as it prevents the close approach of droplets. A higher ionic strength slightly increases particle sizes. This indicates that above the critical salt concentration, attractive forces between droplets including van der Waals and hydrophobic interactions dominate electrostatic repulsion. This results in the aggregation of droplets. Similarly, some authors concluded that adsorbed proteins could retard the flocculation and coalescence of oil droplets at low ionic strength where steric or electrostatic repulsions are predominant (de la Fuente et al., 2002; McClements, 2004).

**TABLE 4:** Effect of process stresses on Dz, polydispersity, zeta potential, and retention rate of curcumin at curcumin loaded‐nanoemulsion stabilized with native and extruded whey protein concentration.

Samples	Dz (nm)		PDI		Zeta potential (mV)		Retention rate of curcumin (%)	
	NWPC	EWPC	NWPC	EWPC	NWPC	EWPC	NWPC	EWPC
Effect pH ionic strength								
0.1	208.4 ^Ab^ ± 2.2	180.6 ± 2.3^Bb^	0.21 ± 0.01^Aa^	0.19 ± 0.02^Aa^	−13.3 ± 0.1^Ba^	−18.6 ± 0.3^Aa^	—	—
0.5	216.8 ± 1.5^Aa^	193.2 ± 2.4^Ba^	0.21 ± 0.02^Aa^	0.20 ± 0.01^Aa^	−9.2 ± 0.1^Bb^	−14.2 ± 0.2^Ab^	—	—
1	219.3 ± 3.2^Aa^	197.2 ± 2.2^Ba^	0.21 ± 0.01^Aa^	0.20 ± 0.01^Aa^	−5.3 ± 0.2^Bc^	−8.1 ± 0.1^Ac^	—	—
Effect of heating								
63°C/30 min (pasteurization)	179.8 ± 1.1^Ab^	143.1 ± 1.7^Bab^	0.24 ± 0.02^Aa^	0.20 ± 0.02^Ba^	−12.4 ± 0.3^Bb^	−18.6 ± 0.2^A^	62.2 ± 0.2^Bc^	68.7 ± 0.3^Ab^
95°C/10 min (boiling)	191.1 ± 1.8^Aa^	147.9 ± 2.3^Ba^	0.24 ± 0.02^Aa^	0.21 ± 0.01^Aa^	−7.5 ± 0.1^Bc^	−14.7 ± 0.1^A^	66.3 ± 0.4^Bb^	73.1 ± 0.4^Ab^
Control nanoemulsion	162.3 ± 2.1^Ab^	136.7 ± 3.1^Bb^	0.20 ± 0.01^Ab^	0.19 ± 0.01^Aa^	−17.7 ± 0.1^Ba^	−23.7 ± 0.1^A^	90.4 ± 0.3^Ba^	94.9 ± 0.2^Aa^
Effect of pH								
pH 3	181.4 ± 1.9^Aa^	149.2 ± 1.5^Ba^	—	—	+7.2 ± 0.1^Bc^	+12.2 ± 0.1^Ac^	—	—
pH 5	165.9 ± 2.6^Ab^	137.5 ± 1.8^Bb^	—	—	−16.3 ± 0.1^Bb^	−22.4 ± 0.2^Ab^	—	—
pH 7	161.5 ± 1.7^Ab^	135.6 ± 1.6^Bb^	—	—	−28.2 ± 0.3^Ba^	−34.5 ± 0.4^Aa^	—	—
Storage stability								
4°C	420.3 ± 2.6^Aa^	218.6 ± 2.4^Ba^	0.38 ± 0.03^Aa^	0.25 ± 0.02^Ba^	−11.6 ± 0.1^Ba^	−18.3 ± 0.2^Aa^	86.5 ± 0.4^Aa^	90.4 ± 0.5^Aa^
25°C	432.5 ± 1.2^Ab^	227.4 ± 3.7^Ba^	0.39 ± 0.02^Aa^	0.26 ± 0.02^Ba^	−9.7 ± 0.1^Bb^	−16.9 ± 0.1^Aa^	78.2 ± 0.2^Ba^	88.1 ± 0.1^Aa^

*Note*: Control nanoemulsion: nanoemulsion containing curcumin stabilized with NWPC (native whey protein concentration) or EWPC (extruded whey protein concentration) at pH 5.50 and temperature of 25°C. Means with the same letter are not significantly different (*p* < .05), capital letters correspond to each row and small letters are related to each column.

Abbreviations: EWPC, extruded whey protein concentrate; NWPC, native whey protein concentrate.

Zeta potential is a parameter that describes the surface charge and electrostatic interactions between particles, which is used to predict emulsion stability. Generally, absolute values greater than 30 mV indicate greater electrical repulsion between particles, which translates into better dispersion (Rao & McClements, 2011). In terms of zeta potential, it was revealed that the increasing ionic strength shifted the zeta potential of all nanoemulsions toward zero. This indicates that the charge on the surface of the protein molecules was reduced upon the addition of salt. This resulted in decreasing electrostatic repulsion and destabilization of the nanoemulsions. However, it was found that the zeta potential of samples containing EWPC was considerably higher than those having NWPC. This suggests that higher ionic strength had a noticeable effect on the instability of the emulsion stabilized with NWPC. In the presence of high ionic strength, salt ions overcome the electrostatic attraction forces between droplets coated with proteins (McClements, 2004), resulting in droplet aggregation. On the other hand, at relatively low concentrations of salt, electrostatic repulsion could dominate the weak van der Waals and hydrophobic interaction, leading to a low tendency toward aggregation (McClements, 2004).

#### 3.6.2 Effect of pH

The impact of different acidic conditions on the particle size distribution and zeta potential of nanoemulsions was determined (Table 4). The Dz for nanoemulsions stabilized with NWPC at pH 3, 5, and 7 was 181, 165, and 161 nm, respectively. It was 149, 137, and 135 nm for samples stabilized with EWPC. Higher Dz observed at lower pH might be correlated with lower absolute net charge, which in turn leads to particle aggregation and increasing Dz. On the other hand, aggregation of particles increases when pH approaches the isoelectric point of whey protein.

In the case of zeta potential, a considerable difference was observed when the pH changed from 3 to 7. Generally, samples stabilized with EWPC represented a higher net charge, implying higher stability. All samples showed a net positive charge at pH 3, related to the fact that below the isoelectric point, the surface charge of the proteins shifts to positive values. No matter which NWPC or EWPC is used, increasing pH led to a negative charge, which increased when pH reached 7. The shift in the zeta potential of the nanoemulsions with changes in pH indicated that the properties of WPC govern the surface charge of the nanoemulsion. In accordance with our results, Rao and McClements (2011) concluded that more stable nanoemulsions were formed at pH 6 and 7 compared to nanoemulsions prepared at lower pH. Those authors reported that at lower pH values, the electrostatic repulsive forces between the particles reduce due to the electrical charge on the surface of the particles, thus leading to particle growth/aggregation (Rao & McClements, 2011). Accordingly, Li et al. (2014) concluded that the net charge of the nanoemulsions stabilized by whey protein isolate containing i‐carrageenan was approximately zero at a pH of 3.66.

#### 3.6.3 Effect of heating

In order to investigate thermal stresses on nanoemulsion stability, pasteurization (63°C—30 min) and sterilization (95°C—10 min) conditions were simulated and the values were compared with the characterization of nanoemulsion at 25°C. At all conditions, the Dz of nanoemulsions stabilized with EWPC was smaller than samples containing NWPC (Table 4). The Dz of the nanoemulsions stabilized with both NWPC and EWPC and was increased under simulated pasteurization and sterilization conditions. However, the changes were sharper for emulsions containing NWPC. Sterilization had a noticeable impact compared to pasteurization conditions. Similarly, Sari et al. (2015) observed that pasteurization conditions slightly increased the particle size, while sterilization conditions led to higher aggregation and thereby increased the particle size. These results were confirmed by PDI analysis. In terms of PDI, it was not significantly changed for samples stabilized with EWPC. Meanwhile, the PDI of samples containing NWPC was increased when imposed to pasteurization and sterilization conditions, implying the broadening of the droplet size distribution. Mustapha et al. (2012) claimed that the hydrophobicity of the protein layer adsorbed on the surface of the droplets and their interactions with each other or with free protein present in the continuous phase correlated with the stability of emulsions. Accordingly, we assumed that the high surface hydrophobicity of EWPC enhances protein‐lipid interactions through hydrophobic interactions and therefore prevents inter‐droplet attraction, which results in fewer droplets aggregating. Moreover, the ability of EWPC to create thick membranes around fat droplets could improve heat stability due to relatively high repulsive steric interactions (Dissanayake et al., 2012; Mozafarpour et al., 2019; Silva et al., 2010). Similar to our results, Çakır‐Fuller (2015) suggested that emulsions prepared with microparticulate WPC were more heat resistant compared with NWPC emulsions. Those authors reported that NWPC stabilized‐emulsion imposed to UHT (140°C) conditions were heat stable at 2% protein concentration, while the emulsions were coagulated at 4% protein concentration, ascribed to inter‐droplet interactions as a result of thermal denaturation. Microparticulated WPC stabilized emulsions, however, were stable at 4% protein levels. It seems pre‐denatured and aggregated proteins break up during emulsion preparation following shear stress, resulting in higher heat stability. Mustapha et al. (2012) showed that the consistency of emulsions prepared with NWPC was increased upon heating at a temperature ≥70°C for 20 min and created aggregated solid‐like pastes/gels. However, emulsions prepared with pre‐denatured WPC showed superior stability against heating, originating from an already denatured state of the protein. Actually, in the case of emulsions stabilized with NWPC, it was suggested that denaturation of adsorbed protein on the surface of the droplets led to cross‐linking of proteins through non‐covalent interactions and hence formed protein network. Therefore, fat droplets were incorporated into the network, resulting in larger particle sizes and lower stability (Dickinson & Parkinson, 2004).

Zeta potential values were shifted toward zero when submitted under pasteurization and sterilization conditions. At all treatments, EWPC‐stabilized nanoemulsions had higher zeta potential values (Table 4). Sari et al. (2015) observed that the zeta potential of WPC‐70 appreciably approached zero after pasteurization and boiling treatments. Those authors suggested that the denaturation of whey protein changes the surface charge of particles.

The retention rate of entrapped curcumin assesses the potential of carrier systems to protect curcumin from degradation. Curcumin is unstable when exposed to environmental stresses. The current study attempts to understand the extent of curcumin degradation under simulated pasteurization and sterilization. Our study revealed that the degradation rate of curcumin was increased when nanoemulsion stabilized with NWPC or EWPC was subjected to simulated pasteurization and sterilization conditions. The highest curcumin retention rate (%) was found in control samples stabilized with EWPC (Table 4). Similarly, Chen et al. (2014) observed that encapsulated curcumin exposed to the UHT process was significantly degraded. The degradation process was found to be more extensive during pasteurization than during sterilization, possibly due to the longer time of pasteurization (30 min) as compared to sterilization (10 min). Curcumin degradation was retarded more effectively in samples stabilized with EWPC, suggesting the coating of EWPC enhanced curcumin protection.

#### 3.6.4 Storage stability

Storage stability determines the suitability of the nanoemulsion for application in the food and beverage industries (McClements, 2004). The stability of nanoemulsions during 1 month was determined at 4°C and 25°C (Table 4). The Dz and PDI of emulsions prepared with NWPC were drastically increased (*p* < .05). In other words, it was almost 2‐fold larger than emulsions prepared using EWPC, indicating that the latter emulsions were more stable during storage. The emulsions kept at 4°C represented slightly lower Dz and PDI compared to samples stored at 25°C. Ma et al. (2017) also reported that the larger particles were created in emulsions stabilized with different surfactants (Tween‐80, lecithin, acacia powder, and whey protein) when samples were stored at higher temperatures, originating from aggregation of particles during the storage process. Those authors noted that particle size decreased after storage due to droplet aggregation and precipitation. Our results were confirmed by Nejadmansouri et al. (2016) who found that the particle size of the emulsion stabilized with WPI increased during the storage period. According to those authors, emulsion stability was affected by two factors: (a) the ability of viscoelastic interfacial films to resist mechanical stresses; and (b) the intensity of steric or electrostatic repulsions. Therefore, nanoemulsion stabilized with EWPC showed relatively high stability against flocculation and coalescence when stored for 1 month at 4°C and 25°C. Meanwhile, the flocculation of droplets in NWPC stabilized emulsion through non‐polar groups is the reason for the aggregation of globular proteins and increasing their particle size during storage (de la Fuente et al., 2002; McClements, 2004).

The zeta potential results confirmed the particle size trend. The results showed that storage time resulted in a significant decrease in zeta potential values. There was a considerable difference between samples prepared with NWPC and EWPC. Meanwhile, it was found that the storage temperature of 25°C is more effective at reducing the zeta potential of nanoemulsions. The higher zeta potential of EWPC‐stable samples indicates their superior stability during storage. Chuacharoen and Sabliov (2019) also found that zeta potential values were significantly reduced when stored at different temperatures.

It was found that EWPC‐stabilized samples exhibited a higher retention rate of curcumin than NWPC‐stabilized samples. While the retention of curcumin in the NWPC nanoemulsion at 4°C was significantly higher than that at 25°C (*p* < .05); there was no difference in the retention of curcumin in the EWPC nanoemulsion at 4°C and 25°C. Accordingly, Joung et al. (2016) reported that curcumin loaded in nanoemulsions was better protected at 4°C than room temperature. This may be due to the fact that curcumin‐nanoemulsion systems are more prone to destabilizing mechanisms such as flocculation and coalescence at higher temperatures. As well, there were no significant differences in the retention rate of curcumin in EWPC‐stabilized samples at 4°C and 25°C, which could be attributed to higher EAI and surface hydrophobicity of EWPC, which facilitate the interaction between WPC and curcumin. Moreover, creating a thicker and viscoelastic layer around curcumin‐oil droplets by EWPC may provide better protection for curcumin at higher temperatures.

### 3.7 Encapsulation efficiency of curcumin

The encapsulation efficiency (EE) indicates the percentage of bioactive compounds that remain after processing, storage, and digestion. EE measures the amount of non‐encapsulated curcumin after centrifugation with respect to the amount used (Ariyarathna & Karunaratne, 2016; Solghi et al., 2020). It is related to particle size, properties of proteins adsorbed on droplet surfaces, and auxiliary processes such as ultrasound, homogenizers, etc. (Ariyarathna & Karunaratne, 2016; Ma et al., 2017; Mohammadian et al., 2019). The EE of nanoemulsion containing NWPC was 90.83 ± 0.3%. However, it was promoted to 95.76 ± 0.2% as a function of the extrusion process. It has also been stated that curcumin EE in protein matrices was approximately 90% (Mohammadian et al., 2019; Solghi et al., 2020). Sari et al. (2015) observed that the loading efficiency of ultrasound‐assisted curcumin‐loaded nanoemulsion in the media composed of medium‐chain triglyceride oil droplets coated with WPC‐70 and Tween‐80 was 90.56 ± 0.47%. Mohammadian et al. (2019) reported that the loading amount of curcumin in the WPI and whey protein microgel was 10% and 90%, respectively.

It seems the extrusion process enhanced EE by improving emulsifying properties, and surface hydrophobicity, and increasing the molecular flexibility of WPC. The extruded WPC effectively encloses the curcumin‐oil droplets due to their flexibility and ability to form thick viscoelastic interfacial layers. Moreover, the high surface hydrophobicity of WPC improves its ability to form a soluble complex with curcumin. Previous studies also suggested that the higher surface hydrophobicity could account for the superior curcumin binding capacity of whey protein (Ariyarathna & Karunaratne, 2016; Gao et al., 2013; Mohammadian et al., 2019; Sari et al., 2015). Furthermore, it seems that emulsion particles might have a significant influence on EE. Emulsion with smaller particles enhances emulsion stability, thus promoting EE. Therefore, smaller particles in emulsions stabilized with EWPC may also increase EE. The current study showed that the EWPC‐based encapsulation system could be used to encapsulate curcumin as a safe and effective delivery system.

### 3.8 In vitro release behavior

**FIGURE 4:**
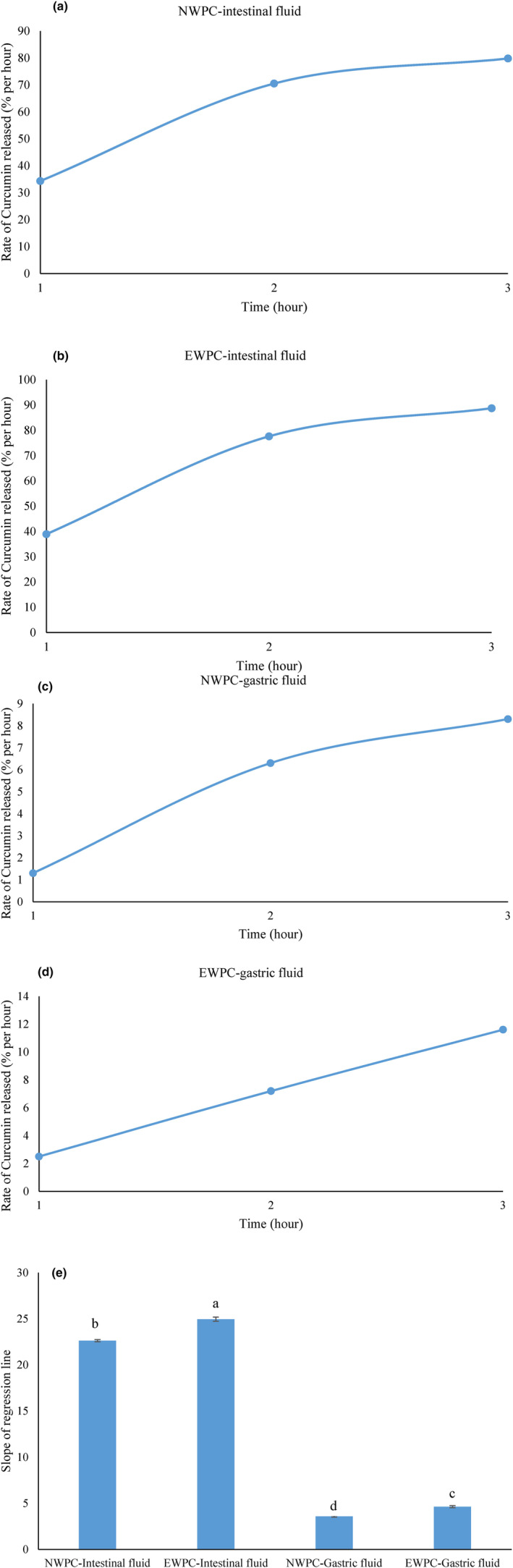
The rate of curcumin released (% per hour) from nanoemulsions stabilized with NWPC (native whey protein concentration) and EWPC (extruded whey protein concentration) at simulated intestinal and gastric fluids during time (a–d). Linear regression (slope of regression line) was used to determine whether there is a significant difference in the rate of curcumin release (% per hour) (e). Means with the same letter are not significantly different (*p* < .05).

In the present study, the rate of curcumin released from nanoemulsion stabilized with NWPC or EWPC under simulated intestinal and gastric conditions was compared. The results are presented in Figure 4. Evaluation of the release behavior of encapsulated bioactive compounds is crucial because it determines their uptake, distribution, and bioavailability. The ability of encapsulation systems to protect against the harsh acidic conditions of the gastric environment is one of their most desirable properties (Mohammadian et al., 2019; Sarkar et al., 2010; Solghi et al., 2020). The slope of regression lines was used to determine whether there are significant differences in the rate of curcumin release from nanoemulsions stabilized with NWPC and EWPC in simulated intestinal and gastric fluids. A burst release was observed in the simulated intestinal condition compared to the gastric environment (*p* < .05). The intestinal liquid contains pancreatin and bile salts, which bind at the oil–water interface, thereby promoting lipase performance in releasing curcumin (Sari et al., 2015). Accordingly, Sarkar et al. (2010) proposed that bile salts enhance the lipolytic activities of pancreatin to breakdown the β‐lg adsorbed at the interface of droplets, which in turn destabilizes the emulsion through displacing the proteins present in the oil–water interface resulting in coalescence of droplets. In addition, the adsorbed pancreatin enzyme promotes proteolysis of the adsorbed protein layer alongside lipolysis caused by the presence of trypsin‐chymotrypsin fractions, which are favored by the bile salts, thereby reducing the surface viscosity of coated proteins and enhancing coalescence. Similarly, Joung et al. (2016) stated that nanoemulsions containing curcumin could destabilize the intestinal environment due to the digestion of β‐lg in the presence of pancreatin.

The results showed that almost 90% (88.7%) of curcumin was released from nanoemulsion stabilized with EWPC in simulated intestinal conditions after 3 hours. Meanwhile, at the same time, almost 80% (82.8%) of curcumin was liberated from nanoemulsion stabilized with NWPC. Similar release behavior has been observed for nanoemulsion samples stabilized with NWPC in simulated intestinal conditions (Mohammadian et al., 2019; Sari et al., 2015). It is possible that the higher percentage of curcumin released from the former samples (*p* < .05) is due to their higher solubility. Accordingly, Ariyarathna and Karunaratne (2016) demonstrated that the higher release of curcumin encapsulated in chickpea protein at pH 2 compared to pH 4 is related to their higher solubility. They suggest that when protein solubility is low, curcumin remains trapped in the matrix and its release is reduced. However, higher solubility allows the encapsulating system to release more quickly. The enhanced release of curcumin may be attributed to the smaller particle size of nanoemulsions stabilized with EWPC. The present study suggests that the sustained release behavior of encapsulating systems consisting of EWPC can be considered a suitable system for increasing the bioavailability of curcumin in the intestine.

In the case of simulated stomach conditions (pH 2), it was found that encapsulated curcumin was stable (*p* < .05). A higher release rate of curcumin was observed in samples stabilized with NWPC compared to their EWPC counterparts (*p* < .05), suggesting that the former samples were less stable under stomach conditions. It may be that samples stabilized with NWPC have lower release rates, since Guo et al. (1995) state that β‐lg, a main component of whey protein (almost 55%), has high stability against pepsin digestion and prevents its breakdown in gastric conditions, thereby reducing the release of curcumin. However, the cross‐linking of whey proteins through hydrophobic and disulfide bonds may be responsible for the lower release rate in samples stabilized with EWPC. In accordance with our deduction, Vahedifar et al. (2018) concluded that increasing hydrophobic and disulfide bonds as a function of the microgelification process reduced the curcumin release rate from the whey protein matrix. The lower release rate from the EWPC matrix indicated a better ability to bind and complex with curcumin. Generally, our study revealed that pretreatment of WPC through extrusion cooking boasted the release of curcumin in simulated intestine conditions. However, a lower release rate was found in the simulated stomach conditions compared to samples stabilized with NWPC. Therefore, the current study indicated that it is possible to control the release of curcumin, which can deliver curcumin to the intestinal tract without notable release in the stomach condition, indicating the expansion of its use in the delivery of bioactive compounds and drugs.

## 4 CONCLUSION

As a hydrophobic and unstable bioactive component, curcumin is difficult to incorporate into aqueous food systems. Therefore, curcumin was encapsulated using a modified WPC‐based nanoemulsion delivery system. The current study revealed that extrusion process effectively increased emulsification ability and encapsulation efficiency of WPC. It was concluded that curcumin was slowly released from the nanoemulsion under simulated digestion, implying that the newly developed nanoemulsion of curcumin has implications for commercial applications. This study demonstrated that using EWPC to deliver highly lipophilic and unstable curcumin based on a nanoemulsion platform is an effective way to improve hydrophilicity, bioavailability, controlled delivery, and degradation resistance.

## AUTHOR CONTRIBUTIONS

**Morteza Jafari:** Conceptualization (equal); data curation (equal); formal analysis (lead); investigation (lead); methodology (equal); software (equal); writing – original draft (lead). **Karim Parastouei:** Conceptualization (equal); data curation (equal); funding acquisition (equal); investigation (equal); project administration (lead); software (equal); supervision (lead); validation (lead); writing – review and editing (lead). **Sepideh Abbaszadeh:** Funding acquisition (equal); investigation (equal); project administration (equal).

## FUNDING INFORMATION

This work was supported by Health research center, Life Style Institute, Baqiyatallah University of Medical Sciences, Tehran, Iran.

## CONFLICT OF INTEREST STATEMENT

The authors declare that they have no conflicts of interest.

## Data Availability

Data available on request with corresponding author.
